# Determination of Tire Wear Particle-Type Polymers by Combination of Quantitative Nuclear Magnetic Resonance Spectroscopy and Soxhlet Extraction

**DOI:** 10.3390/molecules29245899

**Published:** 2024-12-13

**Authors:** Marcel Günther, Gizem Kirimlioglu Sayilik, Wolfgang Imhof

**Affiliations:** Institute of Integrated Natural Sciences, University Koblenz, Universitätsstr. 1, 56070 Koblenz, Germany; maguenther@uni-koblenz.de (M.G.);

**Keywords:** quantitative NMR spectroscopy, microplastic, Soxhlet extraction, natural rubber, styrene–butadiene–copolymer, polyethylene–polypropylene–copolymer, polybutadiene

## Abstract

Tire wear particles (TWPs) are among the most relevant sources of microplastic pollution of the environment. Nevertheless, common analytical methods like IR and Raman spectroscopy are highly impaired by additives and filler materials, leaving only thermogravimetric methods for chemical analysis of TWPs in most cases. We herein present quantitative NMR spectroscopy (qNMR) as an alternative tool for the quantification of the polymeric material used for the production of tires, including natural rubber (NR), styrene–butadiene–copolymer (SBR), polyethylene–co-propylene (EPR) and polybutadiene (BR). Limits of quantification (LOQ) between 3 µg and 43 µg per sample and recovery rates of 72–92% were achieved for all tested polymer types. The first results of combining these measurements with Soxhlet extraction as a sample preparation tool are presented alongside the qNMR experiments.

## 1. Introduction

The abundance of microplastic particles (MPs) is a well-known issue for most environmental scientists. However, recent research gave rise to the fact that the majority of MPs might originate from tires and roads. In 2021, Brahney et al. published a study indicating that up to 84% of airborne MPs stem from this source, mainly deriving from resuspended road dust [1]. Depending on the study, around 0.2–5.5 kg per year and per capita are estimated as global emissions [2]. At the same time, different from regular MP, polymers usually form various kinds of agglomerates at the tire–road interface, and thus, the resulting particles only partially consist of rubber material, while the rest are made up of mineral-based road dust and various tire additives [3,4,5]. In consequence, several interchangeable terms for these kinds of particles are in use, sometimes including every type of material incorporated into these particles and sometimes only focusing on the rubber-based material. The most common terms are “tire and road wear particles” (TWRPs) and “tire wear particles” (TWPs or TPs) [2,3,5,6,7]. In the following, we will use the term TWP to describe all forms of TWRPs and TWPs, but will mainly focus on their polymer-based components. Nevertheless, due to this heterogeneity, TWPs and TWRPs are not always considered MPs, and comparing them with regular forms of MPs is difficult due to the additional lack of necessary data [5,8].

TWP analysis or even quantification is therefore much more difficult than for regular MPs. Although traditional standard methods for MP analysis like IR and Raman spectroscopy can be used, they are highly impaired due to irradiation-absorbing filler materials or fluorescent additives [9,10,11]. Hence, most analyses are performed by other common methods like optical identification, which, however, tends to be biased, or more frequently by thermogravimetric methods like pyrolysis coupled with gas chromatography and mass spectrometry (pyr–GC/MS), which often struggle with the selection of appropriate marker substances [2,5,6,9,11]. For this reason, we want to introduce quantitative nuclear magnetic resonance spectroscopy (qNMR) for the quantification of the polymer-based part of TWPs. In recent years, our group and others have shown that qNMR represents a highly useful tool in the quantification of MPs [12,13,14,15,16,17,18,19]. qNMR combines chemical identification with mass-based quantification and, to some extent, the spectral separation of sample components. At the same time, measurements are fast and, in contrast to thermogravimetric, methods non-destructive [9,14].

In addition, sample preparation techniques are a key step in the transfer of measuring solutions spiked with the corresponding polymeric material as model samples to real environmental samples. So far, density separation, optical sorting and, with certain limitations, also chemical extraction are used to separate TWPs from the surrounding matrix [2,6,7]. As discussed earlier, optical identification and separation methods inherit high biases in most cases, which makes them easy to perform but, at the same time, lowers their overall quality. Density separation, on the other hand, is already well known from traditional MP analysis. Various saline solutions are available, depending on the desired density for separation, but with increasing costs and hazards at greater densities [20]. The results are less biased but tend to differ significantly between studies [21]. A common difficulty poses the change in the density of MPs due to weathering and fouling [22]. This issue seems to be even more pronounced in the case of TWPs, as mineral-based constituents significantly alter the polymer-specific density [3]. Therefore, more and more extraction-based methods are introduced in classic MP separation [18,21,23,24]. However, their adaption to TWPs is not as easy, as the, e.g., vulcanization of rubbers hinders proper dissolution in respective solvents. Instead, most often, only leachable proportions are extracted and analyzed [6].

## 2. Results and Discussion

### 2.1. qNMR Validation

The first requirement when measuring multiple polymer types in one common sample by qNMR is finding a suitable solvent, which can dissolve all targeted compounds. Especially, as synthetic polymers vary in their dissolution properties, we performed several preliminary tests for this purpose, leading us to tetrahydrofuran (THF) as our preferred solvent for tire wear-related polymers. In previous publications, we could already demonstrate the suitability of THF for measurements of polystyrene (PS), polybutadiene (BR) and even polystyrene–co-butadiene (SBR) [16]. Since many tire-related polymers like polyisoprene (PIR) and natural rubber (NR) share structural similarities with BR, foremost, the presence of carbon=carbon double bonds, we expected that THF would also work in this experiment. Moreover, these expectations were exceeded when our preliminary tests showed that THF readily dissolves polyethylene–co-propylene (EPR) as well, although their homopolymers polyethylene and polypropylene are known to be generally insoluble at room temperature and atmospheric conditions [24,25,26]. Apart from sole solubility, economic and practical aspects were also considered, leading to the use of non-deuterated THF as the solvent of choice for our qNMR studies on tire-related polymers as it has already been shown that qNMR also works in non-deuterated solvents [15].

A second requirement, when using non-deuterated solvents like THF for qNMR, is selecting an internal standard that is inert to all other compounds within the solution and, at the same time, gives sharp signals that do not overlap with any other peaks. Although we were using hexamethyl disiloxane (HMDSO) for that purpose in our previous research, this time, we expected the HMDSO signal to be too close to other signals originating from EPR [16]. Therefore, we selected *N*,*N*-dimethylformamide (DMF) as an internal standard instead. DMF depicts three signals within the spectra, two singlets at 2.78 ppm and 2.89 ppm and a third one at 7.91 ppm. None of the three signals interfere with any other signal; however, the singlet at 7.91 ppm is less likely to be affected by possible matrix signals in real environmental samples and was therefore selected as the internal standard of our validation experiments. DMF itself also inherits a very high boiling point of 153 °C; thus, evaporation, and by that, changes in its concentration are not expected [27].

**Figure 1:**
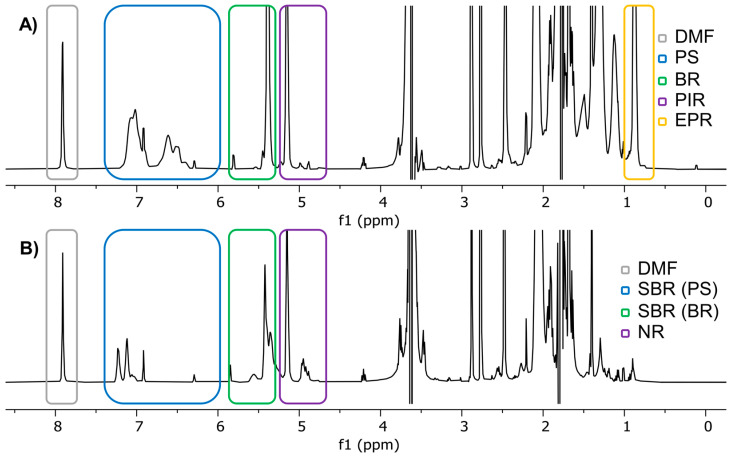
^1^H-NMR spectra of (**A**) sample K_G_1 containing polystyrene (PS), polybutadiene (BR), polyisoprene (PIR) and polyethylene–co-propylene (EPR); (**B**) sample K_N_1 containing polystyrene–co-butadiene (SBR), natural rubber (NR) and *N*,*N*-dimethylformamide (DMF) as an internal standard in tetrahydrofuran (THF). Individual SBR signals are separately labeled as SBR (PS) for styrene units and SBR (BR) for butadiene units.

Figure 1 shows the ^1^H-NMR spectra of two samples containing tire-related polymers. The upper spectrum shows signals for PS, BR, PIR, EPR and, of course, the internal standard DMF, whereas the lower spectrum consists of signals representing SBR (with its components PS and BR) and NR next to DMF. EPR only consists of methylene groups and methyl groups in its propylene units. These groups generate two signals in total, one at 0.87 ppm for the methyl group and one at 1.31 ppm for the methylene group. Theoretically, a third signal for a single proton next to the methyl group could be expected around 1.5 ppm; however, the strong overlap with one or both THF signals impairs any further analysis in this region [28]. Unfortunately, both signals are rather unspecific. In particular, methylene groups occur in almost every other polymer related to tire wear, as well as in many naturally present organic compounds. A precise quantification of ethylene units is therefore not possible. The signal of the methyl groups in the propylene units of EPR, on the other hand, could be used for quantifications if two requirements are met. First, no other polymer must contain a significant number of methyl groups, and second, a proper sample preparation must ensure that no matrix signal overlaps with that signal. However, a small overestimation of the propylene content is therefore expected.

Next to EPR, PIR and NR signals are also shown in the upper and lower NMR spectrum of Figure 1. Usually, both polymers mainly consist of cis-1,4-polyisoprene. The major difference between both polymer types is that NR is a naturally occurring polymer, whereas PIR is its synthetic counterpart. Thus, NR, depending on its source, might contain small traces of other compounds and can vary in its composition [3,29]. In terms of NMR analysis, almost the same signals are expected from NR and PIR. Aside from various methylene and methyl groups, slightly shifted by the nearby double bond, for quantification purposes, the most important signal derives from the single proton attached to the carbon=carbon double bond at 5.15 ppm. Although this signal is very close to the BR-specific signal, they do not overlap due to the neighboring methyl groups in PIR and NR, which shift the signal in NR/PIR to slightly lower ppm values compared to the corresponding signal in BR.

BR, on the other hand, does not contain methyl groups. Fortunately, its signal corresponding to the single proton attached to the carbon=carbon double bond is observed at 5.38 ppm and may therefore be quantified independently from the signals of NR and PIR. Together with aromatic PS signals between 6.30 ppm and 7.20 ppm, BR allows for the quantification of SBR as demonstrated previously [16]. Therefore, PS, although not a typical tire polymer, was included in our investigation.

Comparing the calibration data listed in Table 1, for all selected polymers, a linearity > 0.999 was achieved, at the same time having low RMSD values. These data are in good correlation to calibration data for other types of MPs measured by qNMR. In comparison, Peez et al. (2019) achieved for common MP types, including PS, linearities of 0.994–0.999 at concentrations of 0.03–3.20 mg/mL [12]. Since they are calculated based on the signal-to-noise ratio (SNR), the limit of detection (LOD) and limit of quantification (LOQ) are values mainly depending on the number of scans and the peak shape. Hence, BR and EPR, which both form sharp singlets, result in very low limits in the low µg/mL range. Here as well, Peez et al. (2022) determined a LOD of 1 µg/mL and a LOQ of 2 µg/mL using the same number of scans for PET, a polymer type, which also forms sharp singlets [15]. PS, on the contrary, gives slightly elevated LOD and LOQ values, as the signal for aromatic hydrogen atoms is much broader and therefore results in a lower SNR. If necessary, SNR values may be easily increased by accumulating more scans in each measurement, thus improving the LOD and LOQ as was well described by Dukek et al. [20]. Unfortunately, the SNR will only improve by a factor of $\sqrt{n}$ that way.

As depicted in Table 2, due to the usage of an internal standard for normalization, high precision was achieved in all measurements, aligning with former qNMR studies at values between 99.9 and 98.9% [12,13,14,15,16,17,18]. As the accuracy is given in relative numbers, in general, better values are obtained for greater masses. However, within the calibrated concentration range, all polymer types achieved accuracies between 89.0 and 104.5%, which represent sufficient values for trace analytical purposes.

Table 3 and Table 4 also present separate calibration sets for SBR and NR. Unfortunately, the SBR reference material was of technical quality only, leading to rather imprecise composition data for prepared samples. Hence, any mass difference of PS and BR within the SBR material due to certain filling materials like carbon black or a varying styrene and butadiene ratio would affect calculations by PS or BR calibration data. A similar issue is faced when using PIR calibration data in the quantification of NR, being a natural product showing corresponding impurities of natural origin. Therefore, using the same polymer type for calibration and quantification should avoid imprecise calculations within the extraction analysis depicted in the next paragraph. However, quantification of both polymer types SBR and NR should be possible by either homopolymeric calibrations of PS and BR or calibrations by the use of synthetic variants like PIR. In our previous work, we already demonstrated the use of PS and BR calibrations for SBR quantification [16].

A similar strategy can be used for NR. Table 5 compares the accuracies of the calculated NR and PIR mass of the control samples, either by using the same material for calibration or the respective other one. In general, better results are obtained when using the same material for calibration. Nevertheless, only a difference of around 10% can be observed in using the alternative material for calibration. As explained, the presence of small impurities in NR compared to PIR might account for that difference, as they add to the overall mass of NR material, but not to its NMR signal. This is further underlined, as the masses of NR calculated by PIR calibration data underestimate the actual masses and vice versa.

### 2.2. TWP Extraction

In regard to qNMR measurements, non-deuterated THF has proven to be a suitable solvent for SBR, BR, EPR, PIR and NR. Hence, in the following section, we wanted to test the applicability of qNMR measurements in combination with TWP extraction. For this purpose, we performed a Soxhlet extraction. Other extraction methods, like pressurized liquid extraction (PLE), were considered as well [23,24]. However, in preliminary experiments facilitating these methods, we faced several crucial issues, especially when rubber-like polymers were involved. The exact details are discussed in our most recent study [17]. Like PLE, Soxhlet was also already applied as a sample preparation technique in other MP quantification procedures. For example, Castelvetro et al. extracted polyethylene (PE), polypropylene (PP), polystyrene (PS) and polyethylene terephthalate (PET) in a 3 h Soxhlet process using dichloromethane (DCM) as a solvent to analyze MPs in fishmeal [30]. In our case, THF was selected as it allows for easy handling within the process. After extraction, evaporation of the liquid is necessary to enable reconstitution of the sample in a defined volume of NMR solvent and thus allow for a precise calculation. Hereby, the moderate boiling point of THF facilitates this process. At the same time, the required temperature to maintain a continuous reflux is kept at a minimum. Finally, extraction and measurement can be performed in the same solvent, so the process is not affected by possible differences concerning the solubility of the extracted material.

The extraction process aimed for recovery rates between 80% and 120%, as considered suitable for trace analytics [23]. Unfortunately, this target was only achieved for SBR and BR (Table 6). Both polymer types resulted in recoveries of 87.8–91.5%, which fits well with former studies on MP extraction. In extractions reported by Dierkes et al., recovery rates of 77–118% for PE, 85–95% for PP and 114–131% for PS were achieved [24]. However, they used the pressurized liquid extraction method, which, due to the potential decomposition of rubbers, is not suitable for TWP-related polymers [31]. Results in a narrower range were obtained in the extractions by Castelvetro et al. [30], who observed MP-like PS and PET to be recovered at rates between 95.3% and 106.4%. However, the applied polymer masses were at least twice as high as in our investigations.

EPR, on the other hand, seemed to have just exceeded the acceptable limit for recovery rates (Table 6). In comparison with blank samples, an overlap with impurities—presumably originating from the use of cellulose extraction thimbles—could be observed. In theory, impurities within the sea sand are also a possible source, but since annealed sea sand was used, organic impurities are rather unlikely. Nevertheless, to circumvent this issue, we subtracted the average integral, calculated from the designated region of the EPR signal within the blank samples, from the actual integral of the EPR-containing samples [32,33]. The thus-obtained recovery rate of 90.9% now perfectly meets the requirements. Unfortunately, the method is not straightforward and poses the risk of miscalculations, especially as the quantity of impurities and thus their signal intensity might change depending on the applied thimbles. Further optimization of the EPR extraction conditions is therefore necessary in our future work. The addition of a prewashing step for the thimbles or the use of other thimble materials like glass fiber are conceivable solutions.

Finally, NR and PIR, although extracted at greater quantities, did not reach the required 80% recovery, but reached 77.2% and 78.3%, respectively. This is surprising, as the former NMR measurements did not show any sign of insufficient solubility in THF. However, similar to BR, both PIR and NR contain various double bonds, which might have crosslinked at elevated temperatures during the extraction [31]. Another reason could be the technical grade of the NR and PIR reference material. Impurities or non-dissolvable constituents of the PIR or NR material would add to the weighed masses but not to the NMR results, thus reducing the calculated recovery rates. The similarity in these rates, however, further underlines their shared chemical structure and, therefore, their highly related properties. Nevertheless, further optimization of the process, including the test for shorter extraction times, as well as the need for certified reference materials in the field of MP and TWP research, will be performed in due course [34].

## 3. Materials and Methods

Materials. Polyethylene–co-propylene (EPR) containing approximately 39% propylene, polystyrene–co-butadiene (SBR) containing 23.5% styrene, polybutadiene (BR) and polyisoprene (PIR) containing 96% cis-1,4-polyisoprene were provided by WAGU Gummitechnik GmbH, Warstein, Germany. Natural rubber (NR) also containing 96% cis-1,4-polyisoprene was provided by Weber & Schaer GmbH & Co. KG, Hamburg, Germany. Polystyrene (PS) was supplied by Kissenwelt, Germany. EPR, SBR, BR, PIR and NR were cut from bulk into particles of around 2.5 mg. Polystyrene was present as expanded beads of 0.5–1 mm in diameter. Tetrahydrofuran (THF) ≥ 99.5% was purchased from Carl Roth GmbH & Co. KG, Karlsruhe, Germany. Hexamethyl disiloxane (HMDSO) 99.7% (NMR grade) and *N*,*N*-dimethylformamide (DMF > 99%) were purchased from Thermo Fisher Scientific Inc., Waltham, MA, USA. Extraction thimbles (MN 645, cellulose) were purchased from Macherey-Nagel GmbH & Co. KG, Düren, Germany.

Calibration samples. Two different fractions were examined. The first one contained PS, BR, EPR and PIR. The second one contained NR and SBR. As an NMR solvent, THF containing 0.1% DMF was utilized. For each fraction, five calibration samples were prepared from stock solutions at concentration ranges, as listed in Table 1. Additionally, three control samples were produced for both compositions by dissolving a previously weighed amount of polymer in 1 mL NMR solvent.

Soxhlet process. SBR, BR, PIR, NR and EPR were weighed separately into extraction thimbles at masses of approximately 2.5 mg and mixed into 0.2 g of annealed sea sand acting as a model for an inorganic matrix. For the extraction process, a thimble was then placed into a Soxhlet apparatus before adding 50 mL THF as the extraction solvent. The process was kept under reflux for 24 h. Afterwards, the extract was reduced to approximately 5 mL in volume using a rotary evaporator, followed by being transferred into 20 mL glass vials. The remaining solvent was then evaporated at 60 °C under a continuous stream of air. Thus, the precipitated polymers were again dissolved in 1 mL of NMR solvent for measurement. Three blind samples containing only sea sand, but no polymers, were treated the same way.

NMR measurements. Measurements were performed at room temperature on a JEOL^®^ 500 spectrometer with a 500 MHz 5 mm TH ATM probe head. For all samples, an automated ^1^H WET (target: THF; signals: 2) sequence provided by JEOL was selected, using the following parameters: flip angle = 90°, number of scans = 16, acquired size = 32768, spectral width = 15 ppm, receiver gain = fixed at 42, relaxation delay = 15 s, acquisition time = 4.36767 s. For each sample, 700 µL was added to respective NMR tubes, being measured three times.

Data processing. NMR spectra were processed within the software “MestReNova” [35]. Spectra were referenced to the most shifted DMF signal at 7.91 ppm. Phase correction and baseline correction were manually performed. Apodization was set to 1 Hz. Integrals were determined within the following regions: 0.77–0.97 ppm for EPR (CH_3_), 5.02–5.18 ppm for PIR, 5.04–5.26 ppm for NR, 5.27–5.50 ppm for BR, 6.32–7.40 ppm for PS, 5.19–5.47 ppm for SBR (BR), 6.95–7.32 ppm for SBR (PS) and 7.73–8.07 ppm for DMF. Further calculations were performed in “Excel”. Integrals were normalized and used for calculating respective linear regressions according to the method described by Peez et al. (2019) [12]. Accuracy is expressed as bias by dividing the calculated mass determined by NMR by the initially weighed mass for all model samples. Precision is expressed as relative standard deviation based on three repetitive measurements of the model samples as well. The LOD and LOQ were determined based on the respective SNR in analogy to the procedure from Peez et al. (2019) [12].

## 4. Conclusions

With this research, we were able to show the applicability of qNMR in TWP analytics, enabling the quantification of up to five different types of polymers dominant in this special material. The technique not only allows for the quantification of environmentally relevant quantities but also is unaffected by most additives, which would hinder common TWP analyzing methods. For example, carbon black, usually blocking any infrared radiation, does not create any proton NMR signal. In the same way, fluorescence properties, typically hampering, e.g., Raman spectroscopic quantification of MPs, is not relevant in NMR measurement. Accuracy and precision as well as LOD and LOQ data were observed at values that are suitable for trace analytics. A critical requirement still is the dissolution of the polymeric material, especially of NR, BR and/or PIR. Thus, further research on devulcanization or, likewise, controlled cleavage of crosslinking groups is necessary to bring the qNMR of TWPs to its full potential.

In addition, the first insights into the combination of TWP extraction and qNMR were presented—from our perspective, this is an ideal method for matrix removal and sample preparation. For most selected polymer types, sufficient extraction rates between 87.8% and 91.5% were achieved, with only the polyisoprene-containing types being slightly below an 80% recovery rate. However, we discussed promising ways of further optimization; thus, sufficient extraction should be possible.

## Figures and Tables

**Table 1:** Calibration data for PS, BR, PIR and EPR in THF, including linearity expressed as R^2^, root mean square deviation RMSD, limits of detection (LOD) and limits of quantification (LOQ).

MP Type	Range c [mg/mL]	Linearity R^2^	RMSD	LOD [µg/mL]	LOQ [µg/mL]
PS	0.30–1.51	0.99994	0.00002	12.84	42.80
BR	0.50–2.49	0.99995	0.00003	1.87	6.23
PIR	0.50–2.50	0.99983	0.00002	5.37	17.89
EPR	0.20–0.98	0.99952	0.00016	1.09	3.63

**Table 2:** Control sample data for PS, BR, PIR and EPR, including relative accuracy as bias and precision as relative standard deviation of three repetitions. Mass_grav._ represents the weighed mass and Mass_calc._ represents the mass determined by qNMR.

MP Type	Mass_grav._ [mg]	Mass_calc._ [mg]	Accuracy [%]	Precision [%]
PS	1.34	1.32	98.6	99.8
	1.02	1.00	98.0	99.7
	0.46	0.48	104.5	99.9
BR	2.42	2.35	97.3	99.8
	1.46	1.41	96.5	99.8
	0.85	0.82	96.7	99.9
PIR	2.20	2.15	97.6	99.3
	1.68	1.64	97.3	99.0
	0.65	0.62	95.9	98.9
EPR	0.85	0.83	97.3	99.9
	0.57	0.56	97.6	99.1
	0.27	0.24	89.0	99.4

**Table 3:** Calibration data for SBR separated for its styrene and butadiene subunits and NR in THF, including linearity expressed as R^2^, root mean square deviation RMSD, limits of detection (LOD) and limits of quantification (LOQ).

MP Type	Range c [mg/mL]	Linearity R^2^	RMSD	LOD [µg/mL]	LOQ [µg/mL]
SBR (PS)	0.12–0.59	0.99956	0.00004	23.09	76.97
SBR (BR)	0.38–1.91	0.99979	0.00010	8.89	29.63
NR	0.50–2.50	0.99830	0.00047	5.11	17.04

**Table 4:** Control sample data for SBR separated for its styrene and butadiene subunits and NR, including relative accuracy as bias and precision as relative standard deviation of three repetitions. Mass_grav._ represents the weighed-in mass and Mass_calc._ represents the mass determined by qNMR.

MP Type	Mass_grav._ [mg]	Mass_calc._ [mg]	Accuracy [%]	Precision [%]
SBR (PS)	0.38	0.38	98.7	99.2
	0.59	0.58	98.4	99.7
	0.57	0.56	97.8	99.9
SBR (BR)	1.23	1.19	96.4	98.5
	1.91	1.86	97.3	99.3
	1.84	1.78	96.5	99.7
NR	2.34	2.29	97.7	99.4
	1.74	1.73	99.2	99.7
	0.81	0.80	99.2	99.5

**Table 5:** Accuracy as bias and Mass_calc._ for control samples of NR and PIR, calculated by calibration data obtained from the same reference material (_orig._) or the respective other reference material (_alt._), meaning PIR control samples Mass_calc.,alt._ are based on NR calibration data and vice versa. In addition, the initially weighed Mass_grav._ is given.

MP Type	Mass_grav._ [mg]	Mass_calc.,orig._ [mg]	Accuracy_orig._ [%]	Mass_calc.,alt._ [mg]	Accuracy_alt._ [%]
NR	2.34	2.29	97.7	2.10	89.7
	1.74	1.73	99.2	1.60	91.8
	0.81	0.80	99.2	0.74	91.2
PIR	2.20	2.15	97.6	2.33	110.3
	1.68	1.64	97.3	1.75	109.6
	0.65	0.62	95.9	0.68	109.8

**Table 6:** Extraction data of SBR (separately for PS and BR repeating units), BR, NR, PIR and EPR (EPR* representing the values corrected by blind sample data) given as recovery rate, calculated by dividing the mass determined by qNMR (Mass_calc._) by the mass weighed in the extraction thimble (Mass_grav._), as well as precision expressed as relative standard deviation based on three repetitive measurements.

MP Type	Mass_grav._ [mg]	Mass_calc._ [mg]	Recovery Rate [%]	Precision [%]
SBR (PS)	0.57	0.52	91.3	98.4
SBR (BR)	1.86	1.63	87.8	99.8
BR	2.46	2.25	91.5	99.2
NR	2.47	1.91	77.2	98.7
PIR	2.47	1.93	78.3	99.5
EPR	0.96	1.15	120.1	94.1
EPR*	0.96	0.87	90.9	92.4

## Data Availability

The original contributions presented in this study are included in the article/Supplementary Materials; further inquiries can be directed to the corresponding author.

## Supplementary Materials

The following supporting information can be downloaded at: https://www.mdpi.com/article/10.3390/molecules29245899/s1, Table S1: Concentrations of calibration and control samples for EPR, PIR, BR and PS in THF; Table S2: Absolute intensities of calibration and control samples of EPR, PIR, BR and PS; Table S3: Concentrations and absolute intensities of calibration and control samples of NR; Table S4: Concentrations and absolute intensities of calibration and control samples of SBR; Table S5: Weighed-in concentrations and absolute intensities of extraction samples of EPR, BR, PIR, NR and SBR.
