# Supplementary material for: Determination of Tire Wear Particle-Type Polymers by Combination of Quantitative Nuclear Magnetic Resonance Spectroscopy and Soxhlet Extraction
Source: Molecules. 2024 Dec 13;29(24):5899. doi: 10.3390/molecules29245899 (PMC11679811; doi:10.3390/molecules29245899)
Supplement: Supplementary file 1 [file molecules-29-05899-s001.zip › molecules-3285303-supplementary.pdf]

Supporting information:

Determination of tire wear particle-type polymers by quantitative nuclear magnetic resonance spectroscopy

Marcel Günther, Gizem Kirimlioglu Sayilik, Wolfgang Imhof

Table S1: Concentrations of calibration and control samples for polyethylene-co-propylene (EPR), polyisoprene (PIR), polybutadiene (BR) and polystyrene (PS) in THF.

Sample	$c_{EPR(PP)}$ [mg/ml]	c_{PIR} [mg/ml]	c_{BR} [mg/ml]	c_{PS} [mg/ml]
K_G1	2.50	2.50	2.49	1.51
K_G2	2.00	2.00	1.99	1.21
K_G3	1.50	1.50	1.49	0.91
K_G4	1.00	1.00	1.00	0.60
K_G5	0.50	0.50	0.50	0.30
T_G1	2.18	2.20	2.42	1.34
T_G2	1.47	1.68	1.46	1.02
T_G3	0.70	0.65	0.85	0.46

Table S2: Absolute intensities of calibration and control samples of EPR, PIR, BR and PS as well as the corresponding internal standard (INST).

Sample	I_{INST}	$I_{EPR(PP)}$	I_{PIR}	I_{BR}	I_{PS}
K_G1	0.5989	3.3425	1.6055	3.9274	3.1818
	0.3005	1.6788	0.8103	1.9714	1.5926
	0.2996	1.6635	0.8025	1.9689	1.5894
K_G2	1.7581	8.0091	3.7632	9.2562	7.5269
	0.2838	1.3088	0.6161	1.4864	1.2181
	0.2837	1.2944	0.6178	1.4907	1.2116
K_G3	0.6019	2.1596	0.9964	2.4115	1.9824
	0.3017	1.0948	0.4977	1.2071	0.9959
	0.3012	1.1020	0.4997	1.2055	0.9958
K_G4	1.8020	4.4734	1.9689	4.8589	4.0861
	0.2889	0.7586	0.3231	0.7810	0.6503
	0.2886	0.7553	0.3335	0.7852	0.6597
K_G5	1.7753	2.7254	1.0338	2.4935	2.2036
	0.2852	0.4348	0.1658	0.4019	0.3559
	0.2849	0.4211	0.1609	0.4006	0.3532
T_G1	1.7209	8.3126	3.9928	10.6832	8.0256
	0.2764	1.3331	0.6372	1.7177	1.2925
	0.2766	1.3373	0.6462	1.7235	1.2948
T_G2	1.7321	5.9590	3.0877	6.5249	6.2334
	0.2800	0.9519	0.4890	1.0525	1.0028
	0.2801	0.9469	0.4949	1.0511	1.0100
T_G3	1.8160	3.2638	1.3042	4.0893	3.3608
	0.2913	0.5295	0.2106	0.6569	0.5387
	0.2912	0.5280	0.2138	0.6574	0.5400

Table S3: Concentrations and absolute intensities of calibration and control samples of natural rubber (NR) as well as the corresponding internal standard (INST).

Sample	c_{NR} [mg/ml]	I_{INST}	I_{NR}
K_N1	2.50	1.7439	4.2931
		0.2808	0.6901
		0.2792	0.6879
K_N2	2.00	1.7460	3.5179
		0.2822	0.5671
		0.2794	0.5650
K_N3	1.50	1.8271	2.8000
		0.2935	0.4517
		0.2945	0.4520
K_N4	1.00	1.7792	1.9069
		0.2861	0.3064
		0.2865	0.3067
K_N5	0.50	1.8349	0.9485
		0.2949	0.1518
		0.2949	0.1478
T_N1	2.34	0.5947	1.3562
		0.2981	0.6785
		0.2988	0.6743
T_N2	1.74	0.5502	0.9559
		0.2772	0.4835
		0.2762	0.4822
T_N3	0.81	1.7750	1.4984
		0.2865	0.2393
		0.2863	0.2403

Table S4: Concentrations and absolute intensities of calibration and control samples of polystyrene-co-butadiene (SBR) as well as the corresponding internal standard (INST) integral.

Sample	c_{SBR} [mg/ml]	I_{INST}	$I_{SBR(BR)}$	$I_{SBR(PS)}$
Ks1	2.50	0.5710	2.2997	0.9616
		0.6287	2.4057	1.0656
		0.6440	2.5516	1.0852
Ks2	2.00	0.5725	1.8408	0.8010
		0.6562	2.0993	0.9015
		0.6377	2.0500	0.8817
Ks3	1.50	0.6166	1.4845	0.6506
		0.7027	1.6943	0.7347
		0.6910	1.6720	0.7234
Ks4	1.00	0.6009	0.9979	0.4199
		0.6737	1.1306	0.4796
		0.6552	1.0874	0.4677
Ks5	0.50	0.6027	0.5220	0.2288
		0.6701	0.5799	0.2490
		2.0363	1.7785	0.7609
Ts1	1.61	0.6361	1.5895	0.6996
		0.6810	1.7496	0.7549
		0.6821	1.7135	0.7446
Ts2	2.50	1.0711	4.1242	1.7940
		1.0828	4.2255	1.8089
		1.0674	4.1342	1.7937
Ts3	2.41	1.1520	4.2450	1.8575
		1.1298	4.1787	1.8174
		1.1118	4.0903	1.7914

Table S5: Weighed-in concentrations and absolute intensities of extraction samples of EPR, BR, PIR, NR and SBR as well as the corresponding internal standard (INST) intensity and blank sample data.

Sample	c [mg/ml]	I_{INST}	$I_{Polymer}$
$E_{Blank (EPR)}$	–	0.6412	0.7738
		0.6898	1.2020
		0.6459	0.9157
E_{BR}	2.46	0.6269	3.7498
		0.6198	3.6528
		0.6195	3.6900
$E_{EPR(PP)}$	0.96	0.6271	3.9418
		0.6191	4.3127
		0.6125	3.8701
E_{PIR}	2.47	0.5990	1.2519
		0.6085	1.2739
		0.5988	1.2622
E_{NR}	2.47	0.6060	1.1527
		0.6086	1.1525
		0.5886	1.1417
$E_{SBR(BR)}$	1.86	1.8975	6.4444
		1.8817	6.4053
		0.6029	2.0456
$E_{SBR(PS)}$	0.57	1.8975	2.8823
		1.8817	2.8635
		0.6029	0.8913
